# Editorial: Vegetation resilience in ecological autocatalysis under climate change

**DOI:** 10.3389/fpls.2025.1756384

**Published:** 2026-01-22

**Authors:** Virgil Iordache, Samuel Kuria Kiboi

**Affiliations:** 1 Department of Systems Ecology and Sustainability, and “Dan Manoleli” Research Centre for Ecological Services—CESEC, University of Bucharest, Bucharest, Romania; 2 Department of Biology, University of Nairobi, Nairobi, Kenya

**Keywords:** community resilience, ecological autocatalysis, ecosystem services, plant functional traits, systems ecology, vegetation resilience

The concept of chemical autocatalysis was coined by Wilhelm Ostwald in 1980 (Peng, 2020). Its standard current definition refers to a chemical reaction in which one of the reaction’s products acts as a catalyst. The concept was incorporated into origin-of-life theories in the 1970s and then was adapted by systems ecology to explain the self-organization and growth of ecosystems. There are three main strategies in this interdisciplinary field of research: 1) attempts to reduce the autocatalytic processes in the broad sense to chemical autocatalysis (the reductionist strategy, typical for those working on evolution and the origin of life); 2) investigating the formal mathematical structures (e.g., autocatalytic attractors) distilled from all types of autocatalytic processes (the structural strategy, typical of theoretical biology and ecology, but also applicable to the economy); and 3) approaching each subfield of life science or economics in terms of its specific networks with positive feedback (the autonomist strategy, such as in this Research Topic). We define ecological autocatalysis as a process by which organisms, populations, or communities self-reinforce through circular interactions with their immediate environment, and ecosystems develop circular interactions in which species promote each other, creating positive feedback loops that sustain the resilience of the productive ecological systems at all scales of complexity.

**Figure 1:**
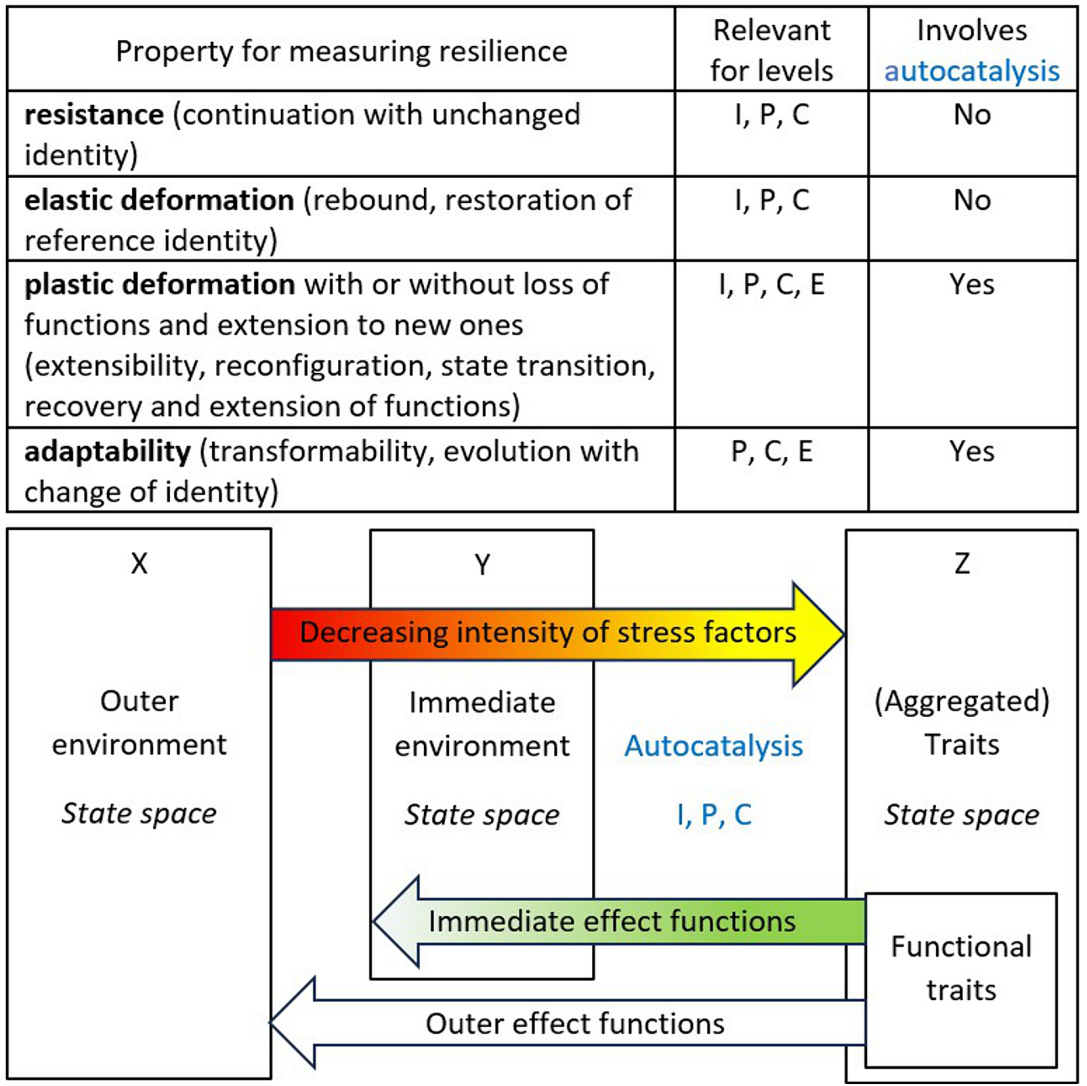
Methodological scheme describing autocatalytic processes relevant for vegetation resilience from individual (I) to population (P) to community (C) levels (adapted from Iordache and Neagoe, 2023). In a broad sense, all these autocatalytic processes are involved in ecological resilience; in a narrow sense, ecological resilience refers only to the resilience of ecosystems (E). We used the X, Y, and Z classes of variables to analyze the articles in this topic.

The topic of “Vegetation Resilience in Ecological Autocatalysis under Climate Change” concerns the roles of the autocatalytic processes depicted in **Figure 1** in shaping vegetation resilience through plastic deformation and adaptability at the individual, population, and community levels and the consequences for ecosystem resilience. We use the term resilience to refer to four properties: resistance, elastic deformation, plastic deformation, and adaptability (Iordache and Neagoe, 2023). We split the environment of plant individuals, populations, and communities into two functional parts: the outer environment and the immediate environment as changed by the biological systems of different complexities, equating the latter with the “extended phenotypes *sensu lato*“ (Diaz, 2025). We identify the roles of autocatalytic processes in vegetation resilience, as shown in **Figure 1**. These processes have lower complexity than the usual ecosystem autocatalytic behavior conceptualized as species with specific functional traits or fluxes of energy and elements (Veldhuis et al., 2018; Jordan, 2022).

Three articles in the Research Topic causally link X variables (the outer environment) to Z variables (the trait’s state space; **Figure 1**). Ge et al. examine the interactions among vegetation, surface water, and climate in the Bosten Lake Watershed, NW China, using EEMD to analyze multi-timescale dynamics, revealing significant correlations and the impacts of climate change. Li et al. assessed the effects of climate change on *Hovenia dulcis* habitats in China using the MaxEnt model, identified key environmental variables, and predicted future distribution shifts under different climate scenarios. Sinan et al. investigated the impact of drought on net primary productivity (NPP) in Inner Mongolia, using the CASA model and the SPEI index to analyze spatial and temporal trends and develop drought-loss rate curves. Such studies at regional and macroregional scales lay the groundwork for investigating vegetation resilience.

Two articles approached the same X-Z causation chain and explicitly assessed resilience in terms of resistance and elasticity. One study examined the stability and sensitivity of gross primary productivity (GPP) to climate variability in China from 1982 to 2019 (Xu et al.). The second one explored carbon sink stability in ecological restoration areas in China, driving mechanisms, and future predictions under different climate and human activity scenarios (Xu et al.). This kind of research enables the investigation of autocatalytic processes that support plastic deformation and the adaptability of vegetation.

Three articles used a Y (immediate environment, **Figure 1**) – Z causation chain. Jin et al. investigated the successional dynamics between alpine meadows and alpine steppes on the Qinghai-Tibet Plateau, focusing on species and functional diversity, community stability, and ecosystem multifunctionality. Ma et al. examined nonstructural carbohydrate allocation in *Spiraea* across Altai Mountain grasslands, highlighting the influence of environmental factors, including temperature, soil water content, and nitrogen, on carbohydrate concentrations and shrub adaptation strategies. Camel et al. investigated the mortality, structure, propagation, and microhabitat of *Haageocereus acranthus* in Peru’s coastal lomas, highlighting human impacts, environmental factors, and conservation needs for this endemic cactus species. This kind of research covers the first part of the autocatalytic cycles depicted in **Figure 1**.

Zhou et al. used the longest causal chain (X to Z to Y) to investigate the effects of warming on leaf light-use efficiency and functional traits in alpine plants through a four-year field experiment, focusing on photosynthesis, leaf traits, and soil nutrients. Xin et al. also examined the effects of Z (independent variables) on Y (immediate environment). They investigated rainfall interception by sand-fixing vegetation and its impact on soil carbon and nitrogen distribution in sand-covered hilly areas, focusing on different vegetation types and their canopy structures. This kind of research covers the second part of the autocatalytic cycles (the immediate effect functions, **Figure 1**).

At the time of writing this editorial, there were 7830 articles with the terms “vegetation” and “resilience” in their titles, abstracts, or keywords in Web of Science; 207 also included the term “functional traits”; only one article had the terms “vegetation” and “autocatalysis”. From 2023, the number of articles published by year increased sharply. An analysis of the citation networks among the 7830 articles using CiteSpace (Chen, 2005-2022) revealed 13 main clusters of literature. Two clusters of literature included the term “vegetation resilience” as a keyword, and one of these also included the term “trait”.

To demonstrate the role of vegetation resilience in ecosystem resilience and its consequences for the production of ecosystem services, stronger cooperation between plant and environmental scientists is needed, combining structural and autonomist strategies to address autocatalytic processes. Such cooperation would be facilitated by a general theoretical background covering all autocatalytic processes relevant to resilience, from organisms to ecosystems, and by specific research infrastructures. Systems ecology is well-positioned to be reconceptualized to address this problem (Iordache, 2025), and successful long-term ecological research sites can provide institutional models for interdisciplinary research (Iordache and Groffman 2025). Specific information, such as the role of large organisms (Vasseur et al., 2025) and the modular structure of trait spaces (Carmona and Beccari 2025), allows the formulation of specific, tractable research hypotheses. The geographical coverage of continents with less intensive research is also a priority, as climate change manifests everywhere (Bedair et al., 2023). Plant scientists and botanists can increase collaboration with ecosystem ecologists and environmental and earth system scientists to address the complex challenges of vegetation resilience under climate change stress using plant functional traits.
